# Enhancing the Early Hydration of Supersulfate Cement: The Effect of Sodium Aluminate

**DOI:** 10.3390/ma18061228

**Published:** 2025-03-10

**Authors:** Jiawei Wang, Ting Li, Jinbang Wang, Chong Zhang, Xiuzhi Zhang, Guangbin Duan

**Affiliations:** 1 School of Materials Science & Engineering, University of Jinan, Jinan 250022, Chinaa3836383@163.com (C.Z.); mse_duangb@ujn.edu.cn (G.D.); 2 China Communications Construction Group Second Engineering Co., Ltd., Jinan 250022, China; 3 Shandong Provincial Key Laboratory of Preparation and Measurement of Building Materials, University of Jinan, Jinan 250022, China; 4 Hock Technology Co., Ltd., Jining 272104, China

**Keywords:** supersulfate cement, sodium aluminate, hydrolysis reaction, ettringite, C-(A)-S-H gel

## Abstract

Supersulfate cement (SSC) has received significant attention in the construction industry due to its extensive utilization of solid wastes and low carbon emissions. However, the low carbonation resistance and early strength of SSC greatly restricted its application, which was attributed to early insufficient alkalinity and slow hydration. Facilitating early hydration alkalinity is critical to promote early hydration and improve early performance for SSC. Thus, sodium aluminate (SA), an admixture with concentrations ranging from 0% to 4%, was adopted to enhance early alkalinity and investigate its impact on the initial hydration process. The results indicated that incorporating SA into SSC enhances its early performance by balancing high alkalinity and AFt stability. The addition of 2% SA accelerates hydration procession, reducing initial and final setting times by 76% and 42%, respectively, while increasing viscosity by 50% for improved structural stability. At 2% SA, 1-day and 7-day compressive strengths rose from 3.7 MPa to 8.4 MPa and from 15.1 MPa to 18.5 MPa, respectively, representing gains of 127% and 22.5%, which were facilitated by accelerated GGBFS dissolution and needle-like AFt formation, which densifies the crystal-gel network microstructure.

## 1. Introduction

Cement is one indispensable material in the traditional construction industry. However, the high energy consumption and significant carbon dioxide emissions during the production process have been contradictory to the development path of green and low-carbon cementitious materials [1]. Thus, it is imperative to develop and research low-carbon cementitious materials [2]. Supersulfate cement (SSC), a promising solution, has gained significant attention for its environmentally friendly and green properties. Generally, it consisted of a ground granulated blast furnace slag (GGBFS) (accounting for about 70–75%); sulfate sources such as flue gas desulfurization gypsum, hemihydrate gypsum, or anhydrite (accounting for 10–20%); and an alkali activator (no more than 10%) [3]. The production of SSC concrete reduces CO_2_ emissions compared to Portland cement (PC) concrete (from 765 kg CO_2_/t to 68 kg CO_2_/t) [4]. In terms of carbon emission and strength, SSC concrete based on volcanic pumice has significant advantages. Compared with PC concrete, the total emissions of SSC concrete with 5% and 15% gypsum content are 172.93 and 193.08 kg CO_2_·eq/m^3^, respectively, which are 54% and 49% lower than PC concrete. Compared with other green concrete, SSC concrete has better 90-day strength than concrete with PC contents of 180 kg/m^3^ and 360 kg/m^3^ fly ash, and the emissions are 10% to 20% less [5]. However, SSC faces challenges related to inadequate carbonation resistance and suboptimal early-stage mechanical properties, which limits its application in practical engineering [6]. Actually, the insufficient hydration of GGBFS and the deficiency in alkalinity are critical factors contributing to the inadequate carbonation resistance of SSC [7]. Therefore, enhancing the early hydration process of SSC is a crucial approach to addressing issues of low early strength and inadequate carbonation resistance.

Research has shown that improving the addition of nano-additives has a positive effect on the early performance of cementitious materials [8]. By adding FA and nano-SiO_2_, Golewski significantly improved the mechanical properties of the ternary cement, which led to a more homogeneous phase system in the cementitious matrix [9], while Deng [10] incorporated the supplementary nano-AFt and found it offered more nucleation sites for AFt formation. It should be made clear that the nucleation effect of nanomaterials and the activation reaction of gelling materials jointly affect the hydration enhancement process of materials. However, the agglomerating effect of the nano-additive hinders particle dispersion, and its high specific surface area consumes excessive water, preventing late strength gain in SSC [11].

In addition, it was found that suitable chemical admixtures control the hydration performance of SSC by regulating alkalinity, which was determined by the dissolution of chemical admixtures. Rubert [12] adjusted the KOH content and found that lower base content favors the generation of C-S-H gel, while higher base content converted AFt into monosulfate. Similarly, Xing [13] adopted sodium citrate and found that low pore solution alkalinity was the key to improving SSC performance in a continuous hydration process. Ultimately, these changes influenced the early performance of SSC. Briefly, the early performance of SSC could be influenced by regulating the alkalinity through chemical admixtures, which affected the GGBFS dissolution and accelerated the hydration process.

Additionally, the early performance could also be influenced by the composition and morphology of hydration phases, especially for the emergence of AFt at an early age [14]. Further, the growth process of AFt is influenced by the concentration of aluminum ions and pH value [15]. Wang [16] adjusted the concentration of sulfate and aluminum ions to gain the AFt crystal seed and improved the early mechanical properties of the cement. In addition, the incorporation of aluminum-rich materials raised the aluminum ion concentration and resulted in a more uniform and regular morphology of AFt crystals [17]. Additionally, Chang points out that the content of ettringite decreased as the initial alkalinity in the pore solution increased [18]. Moreover, Li [19] reported that the optimized pH range for the stable existence of AFt was 10.5 to 13.0, which would be disintegrated when the pH was beyond this range. Based on the above literature, it is not difficult to find that high alkalinity was beneficial for improving the early performance and anti-carbonation resistance of SSC, but it was not favorable to the formation of AFt and the development of early mechanical properties [20]. Therein, sodium aluminate (NaAlO_2_, SA), a salt of strong base and weak acid and rich in aluminum ions, was dynamically dissolved in water to form an amorphous Al(OH)_3_ phase and release OH^−^ [21], which was expected to balance the contradiction between high alkalinity and AFt formation and demonstrated a pronounced effect.

The present study was conducted with a view to enhancing the early performance of SSC and investigated the effects of SA content on multiple parameters, including rheological properties, hydration products, mechanical properties, and the hydration process. Additionally, the action mechanism of SA was analyzed through measurements of hydration heat, ion concentration, and pH values. Furthermore, the composition, morphology, and microstructure of the early hydration products were characterized by X-ray diffraction (XRD), thermogravimetric analysis (TG-DTG), and scanning electron microscopy with energy dispersive spectroscopy (SEM-EDS). The overarching objective of the present investigation is to elucidate the function of SA in the initial hydration process of SSC, providing both theoretical and experimental insights to extend its application in engineering contexts.

## 2. Materials and Methods

### 2.1. Raw Materials

**Figure 1:**
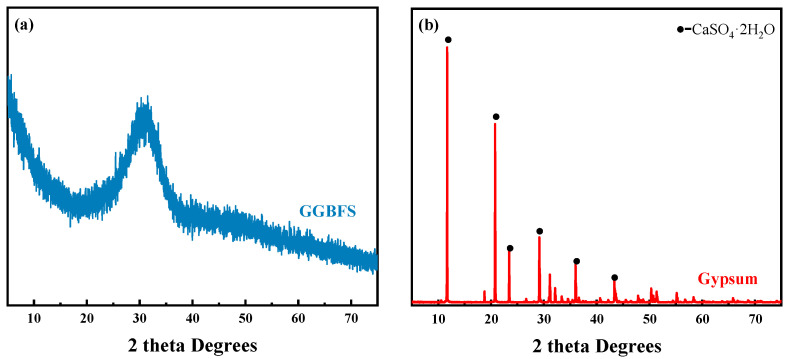
XRD pattern of materials: (**a**) GGBFS; (**b**) gypsum.

**Figure 2:**
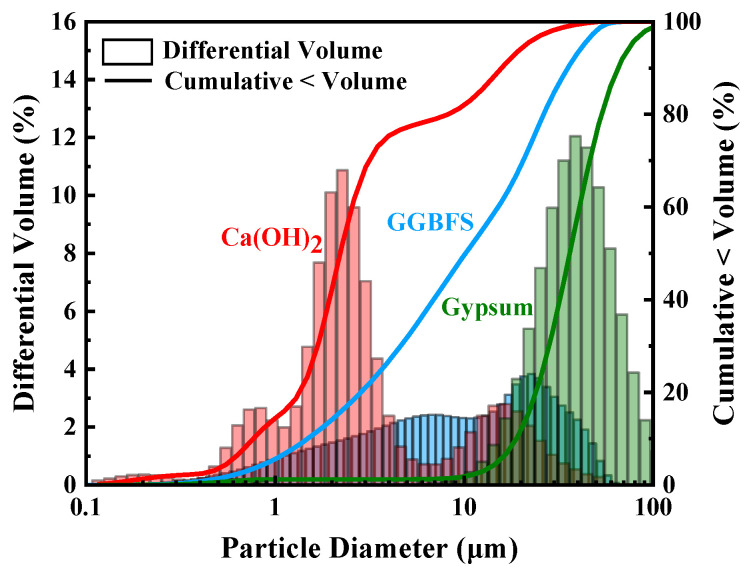
Particle size distribution of raw materials.

The following substances were utilized in the preparation of SSC: GGBFS, dihydrate gypsum, and Ca(OH)_2_. The 28-day activity index for GGBFS was found to be 113%. The chemical composition of the GGBFS and gypsum is detailed in Table 1. Figure 1 displays the XRD pattern of GGBFS and gypsum, indicating that GGBFS contains a lot of amorphous phases and the primary constituent of gypsum is calcium sulfate dihydrate (CaSO_4_·2H_2_O). The particle size distribution of the materials is illustrated in Figure 2. The D50 values of GGBFS, calcium hydroxide, and dihydrate desulfurized gypsum are 9.82 μm, 2.27 μm, and 12.05 μm, respectively. SA with active ingredient contents exceeding 95% was added to regulate the setting time. The SSC prepared in this study conforms to the T/TMAC 046-2022 group standard for gypsum slag-based cement.

### 2.2. SSC Preparation

**Figure 3:**
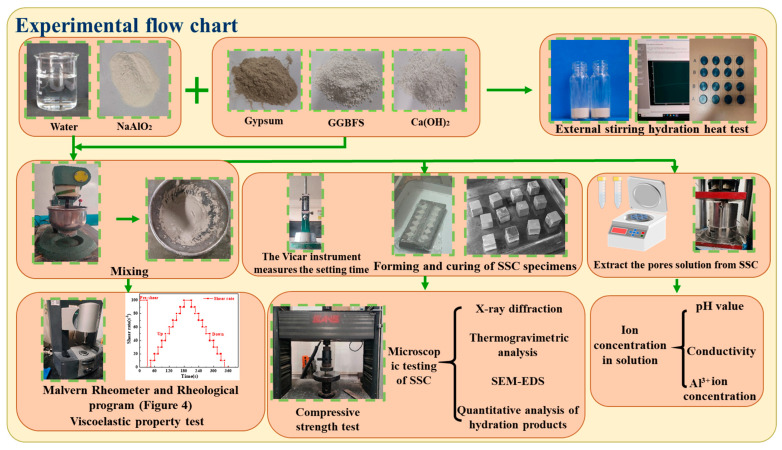
Experimental flow chart.

Figure 3 displays the experimental flow chart. The composition of the reference group SSC is 82.5% GGBFS, 10% gypsum, and 7.5% Ca(OH)_2_. The water-to-binder ratio of the mixture is 0.4. Assuming that all other factors remain unchanged, SA was added to the SSC at dosages of 1%, 2%, 3%, and 4% based on the mass of materials. Before preparing SSC, the weighed SA was dissolved in water and sealed. The solution was left at room temperature for 12 h. During the experiment, the dry powders were stirred for 60 s until homogeneous. The prepared SA solution was subsequently introduced into the mixture to ensure a homogeneous SSC paste. Finally, the paste was poured into the mold, and the rheological properties of the paste were measured. The laboratory temperature was precisely controlled at 20 ± 2 °C.

### 2.3. Test Methods

#### 2.3.1. Setting Time Test

The setting time test was conducted in accordance with the method specified in GB/T 1346-2011 [22]. The paste was transferred into a frustum-shaped container with a glass sheet placed at the bottom to test its setting time. The initial setting time of SSC was established by recording its duration from the moment the powder was blended with water until the Vicat apparatus pointer descended to a position 4 ± 1 mm above the bottom of the container. Then, turn the frustum-shaped container over and continue to measure the final setting time. The measurement was completed at the height that the pointer of the Vicat apparatus dropped, which was less than 0.5 mm.

#### 2.3.2. Compressive Strength Test

The compressive strength experiment is tested with reference to the standard GB/T 17671-2021 [23] and the universal electronic experimental machine, using a mold size of 20 mm × 20 mm × 20 mm [24]. The stirred paste is loaded into a mold, and vibration is used to ensure that all air is expelled. After curing for 1 day, the specimens were taken out of the mold, and then the hardened specimen continued to be cured to the specified age for mechanical property testing. The temperature of the curing room was maintained within a range of 20 ± 2 °C, and the relative humidity was maintained at a level of at least 95%.

When the specimens were cured to the specified age, they were taken out and subjected to compressive strength testing. The rate of load application was 0.6 kN/s, and the mean mechanical property of six samples was determined. Specimens with errors exceeding ±15% of the average value were excluded, and the final compressive strength for the group was calculated based on the mean value of the remaining specimens.

#### 2.3.3. Rheological Performance Test

The objective of this study is to evaluate the rheological properties of SSC paste. To this end, a Malvern rotational rheometer was utilized. Specific test methods include yield stress tests and viscosity tests. SAOS was used to follow the change in energy storage modulus over time, enabling the characterization of the morphological alterations in SSC paste.

1. Yield stress and viscosity test

**Figure 4:**
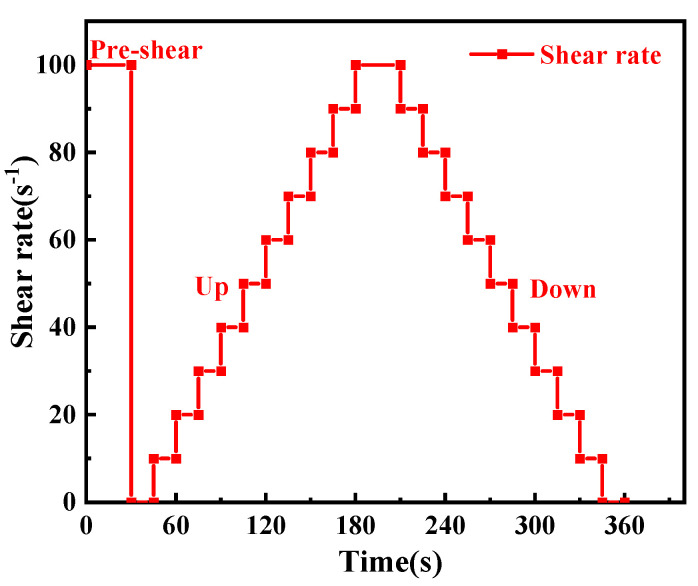
The test procedure of the rheological performance test.

As delineated in Section 2.2, the paste was meticulously transferred to the rheometer plate. First, the paste was subjected to a pre-shearing process for a duration of 30 s at a rate of 100 s^−1^, after which it was allowed to rest for 15 s. Then, the shear rate was divided into 10 segments and reduced from 100 s^−1^ to 0.001 s^−1^ (as indicated in Figure 4). The shear stress at various shear rates is recorded [25]. Then, using the Bingham model (Equation (1)), a curve-fitting analysis was performed [26].

τ = τ_0_ + μ γ (1)

where τ is the shear stress (Pa), γ is the shear rate (s^−1^), τ_0_ is the yield stress (Pa), and μ is the shear viscosity (Pa·s).

2. Small-amplitude oscillatory shear (SAOS)

**Figure 5:**
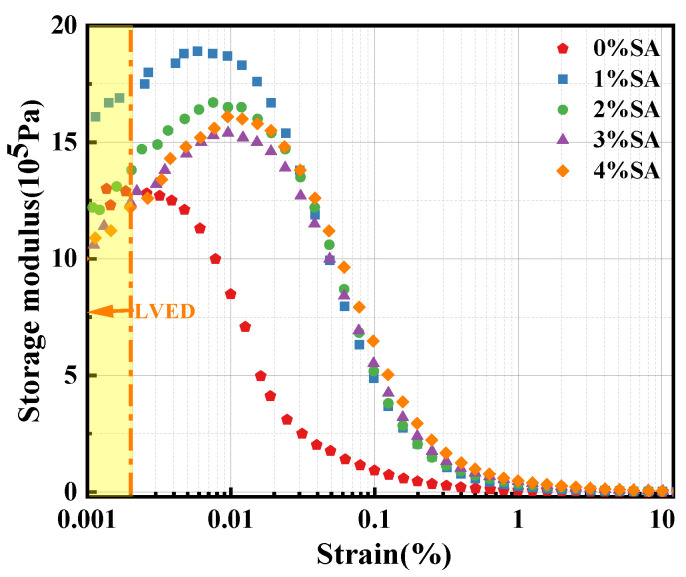
Variation of energy storage modulus of the paste with strain rate.

Research shows that the SAOS test can be utilized to measure the change in the structure of materials at an early age over time because of its non-destructive properties to materials [27]. Before starting the measurement, Malvern rheometer was used to test the change of SSC storage modulus with strain, as shown in Figure 5. When the strain is less than 2 × 10^−5^, the storage modulus of each group increases gradually with the increase in strain, indicating that the material structure is not destroyed by shear. However, when the strain exceeds 2 × 10^−5^, the storage modulus of SSC gradually decreases, indicating that the material structure is damaged due to shear [28]. Therefore, the subsequent control strain is constant at 2 × 10^−5^, and the change of SSC energy storage modulus with time is tested.

After adding water to the cementitious material and mixing for 5 min, the paste was placed on the plate of the Malvern rheometer, with the distance between the plates adjusted to 1.0 mm. A soft film was placed around the plate during the measurement to prevent water evaporation. The limiting strain of the paste is approximately 2 × 10^−5^, as demonstrated in Figure 5. Then, the storage modulus and loss modulus of the SSC paste were tested at a shear strain of 2 × 10^−5^ over a period of 4 h. During the measurement process, data were collected at intervals of 30 s.

#### 2.3.4. Calorimetric Test

The stirred paste was transferred to a glass bottle and then placed in an 8-channel TAM Air calorimeter to determine the hydration heat release of SSC within 24 h. This setup allowed accurate monitoring of the thermal heat released or absorbed during the initial reactions of the SSC samples as they hydrated. The laboratory temperature is 20 °C.

#### 2.3.5. Ion Concentration in Solution

The ion concentrations in the pore solution, including pH, conductivity, and Al^3+^ ion concentration, were tested at different time intervals after the addition of water to the sample. Unhardened samples were centrifuged at 7500 rpm, and hardened samples were extruded using the uniaxial compression steel die method followed by a centrifugal operation to obtain pore solutions. A PHS-3E pH meter (LABO-HUB, Beverly Hills, CA, USA) was used to measure pH at 23 ± 2 °C. A DDS-307A conductivity meter was employed to record conductivity values at different times. The aluminum ion concentration was measured with a spectrophotometer using the colorimetric method according to the procedure of the Chinese standard GB/T 5750.6-2023 [29].

#### 2.3.6. X-Ray Diffraction (XRD)

After mixing raw materials and additives in a mixer, five sets of paste samples were prepared, corresponding to 8 h and 1 day of hydration. Isopropyl alcohol was added to stop the process of sample hydration. The sample is then ground and sieved to obtain a fine powder suitable for further XRD testing. This method allowed for the identification of crystalline phases in SSC. The equipment used for XRD test is D8advance X-ray diffractometer. (The target is a Cu target, the acceleration voltage is 40 kV, the operating current is 40 mA, the 2θ angle ranges from 5° to 80°, and the step size is 0.0200 S^−1^). Finally, physical phase characterization of samples hydrated for 8 h and 1 day was performed using HighScore Plus 3.0e software.

#### 2.3.7. Thermogravimetric (TG) Analysis

TG tests were conducted to investigate the thermal stability and mass changes of the SSC samples under various hydration conditions. Approximately 20 mg of each sample, select SSC without SA, SSC with 2% SA, and SSC with 4% SA, were used for TG analysis. TG test equipment is TGA/DSCI synchronous thermal analyzer. Samples were subjected to heating from 30 °C to 900 °C at a rate of 10 °C/min, ensuring a constant heating rate. The flow rate of the argon gas was set at 50 mL/min to ensure optimal conditions for analysis.

#### 2.3.8. SEM-EDS

The SSC samples whose hydration reactions had been terminated by isopropyl alcohol were dried in an environment at 50 °C. Then, conductive adhesive was used to coat samples on the SEM sample stage. After that, a high-resolution ion coater was employed to plate the samples with gold to enhance their electrical conductivity, facilitating the acquisition of SEM images. SEM test equipment is QUANTA250FEG scanning electron microscope. SEM images were taken of the samples containing 0% SA, 2% SA, and 4% SA hydrated for 1 day, and EDS analysis was conducted on the images of the samples containing 4% SA.

#### 2.3.9. Quantitative Analysis of Hydration Products

**Figure 6:**
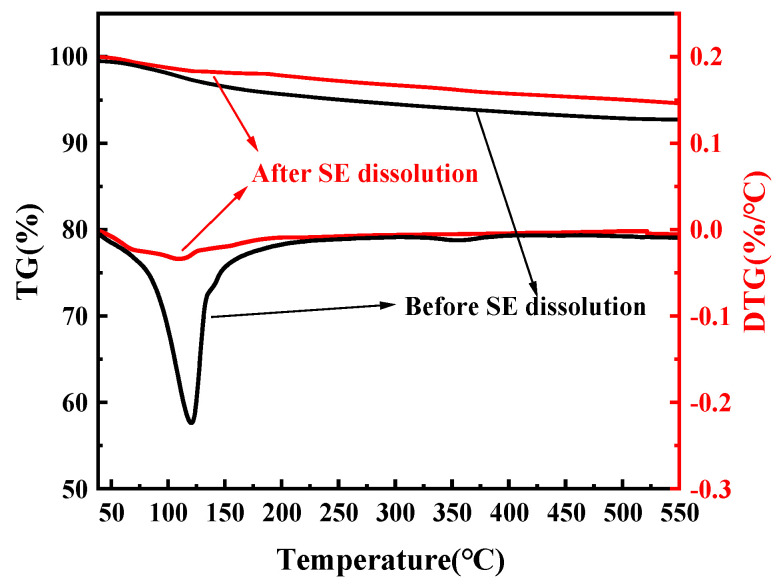
TG/DTG patterns before and after SE dissolution.

The C-(A)-S-H gel has been observed to undergo significant water loss in the temperature range of 50–600 °C, with the predominant mass loss typically occurring at 120 °C. The water loss peak of AFt is also approximately 120 °C, coinciding with the water loss peak of the C-(A)-S-H gel. Therefore, the SE method is utilized to affect the separation. As shown in Figure 6, AFt in the sample is selectively dissolved using 5 wt% Na_2_CO_3_ and dried before thermogravimetric analysis to further analyze the hydration products [30]. EDTA titration test was performed using samples of SSC without SA, SSC with 2% SA, and SSC with 4% SA hydrated for 8 h and 1 day. The degree of GGBFS hydration was evaluated following the procedure detailed in GB/T 12960-2019 [31], utilizing the selective dissolution technique. An EDTA solution of 0.15 mol/L and a NaOH solution of 50 g/L containing 33.3% by volume triethanolamine (TEA) solution were used [32]. The pH was corrected to 11.60 ± 0.05 using NaOH solution, after which 0.30 ± 0.01 g of samples that had been hydrated for 8 h and 1 day were separately added. The samples were agitated with a magnetic agitator for 30 min, washed twice with isopropanol, filtered, and dried to obtain the samples to be tested. The mass percentage of C-(A)-S-H was determined using the EDTA titration and selective dissolution steps. The extent of GGBFS hydration was then calculated based on Equation (2) [33]:

α_s_ = (1 − (100 × R_SSC_)/M_SSC_ × F_GGBFS_ × (100 − W_SSC_)) × 100% (2)

where α_s_ is the degree of GGBFS hydration, R_SSC_ is the dried residue of the SSC after EDTA dissolution (g), M_SSC_ is the mass of SSC paste (g), F_GGBFS_ is the mass fraction of GGBFS in SSC, and W_SSC_ is the chemically bound water content in SSC samples (g/100 g).

## 3. Results

### 3.1. Effect of SA on Workability Performances of SSC

#### 3.1.1. Setting Time

**Figure 7:**
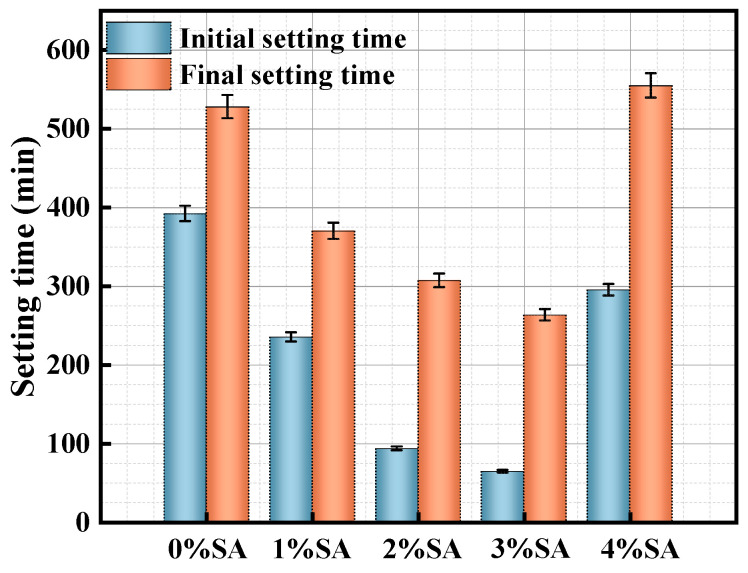
Effect of SA content on the setting time of SSC.

Figure 7 illustrates the setting time of SSC with different proportions of SA included. The SSC without SA had an initial setting time of approximately 400 min and a final setting time of approximately 528 min. With an increase in the SA content, both the initial and final setting times of the SSC decreased. The addition of 1% SA, 2% SA, and 3% SA led to reductions in the initial setting time of SSC by 40%, 76%, and 84%. Similarly, the final setting time was decreased by 30%, 42%, and 50% for these concentrations of SA. The reduction in setting time is probably attributed to the incorporation of SA, as it enhances the alkalinity of the SSC system. Nevertheless, an intriguing phenomenon was observed in the SSC containing 4% SA; the setting time was prolonged relative to groups with lower SA concentrations, warranting additional research to understand this discrepancy.

#### 3.1.2. Viscosity Test

**Figure 8:**
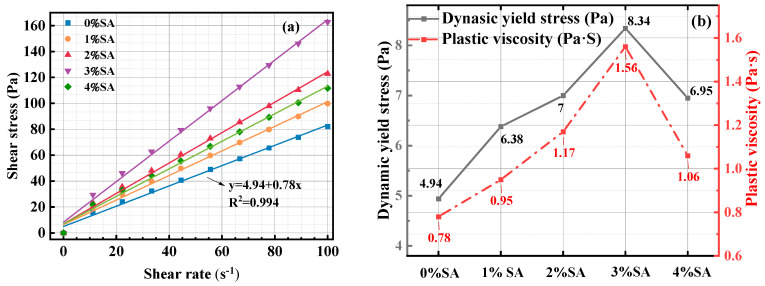
Effect of SA on rheological performance of SSC: (**a**) Bingham fitted curves; (**b**) yield stress and viscosity.

To investigate the impact of SA doping on the rheological characteristics, the viscosity of the SSC was evaluated. Bingham fitted curves for the SSC paste are illustrated in Figure 8a, and Figure 8b shows the rheological parameters of the SSC paste change with different doses of SA, using the data from Figure 8a.

As illustrated in Figure 8b, with the escalation in SA dosage, both the yield stress and viscosity initially rise before subsequently declining. The peak values are attained when the SA concentration is at 3%, where the yield stress and viscosity reach their maximums of 8.34 Pa and 1.56 Pa·s, respectively. Research [34] shows that the SA dissolved in the water partially hydrolyzed to form NaOH and Al(OH)_3_, which promoted the dissolution of GGBFS. Consequently, an augmentation in the ionic content of the pore solution results in an increase in the van der Waals forces between the particles. It has been demonstrated that increasing the alkalinity of the system is conducive to the development of hydration products, such as C-(A)-S-H gel, as illustrated in Equation (3) [35]. This not only consumed free water but also bridged the unreacted solid particles [36]. Consequently, both the viscosity and yield stress exhibit a positive correlation with the increasing content of SA.

2Al(OH)_3_ + Ca^2+^ +SiO_4_^−^ + 2H_2_O → C-A-S-H + OH^−^ (3)

When the SA dosage was 4%, the yield stress and viscosity of SSC were lower than those at 3%. The possible reason is that the addition of a substantial quantity of SA to the paste results in an excessively alkaline environment, which hinders the formation of AFt. The chemical reaction between Al^3+^ and Ca^2+^ led to the generation of calcium aluminate hydrate, which encapsulated raw material particles [37]. This procedure inhibited the breakdown of the raw material, consequently decreasing the usage of free water and resulting in reduced viscosity and yield stress.

#### 3.1.3. Effect of SA on the Storage Modulus of SSC Paste

**Figure 9:**
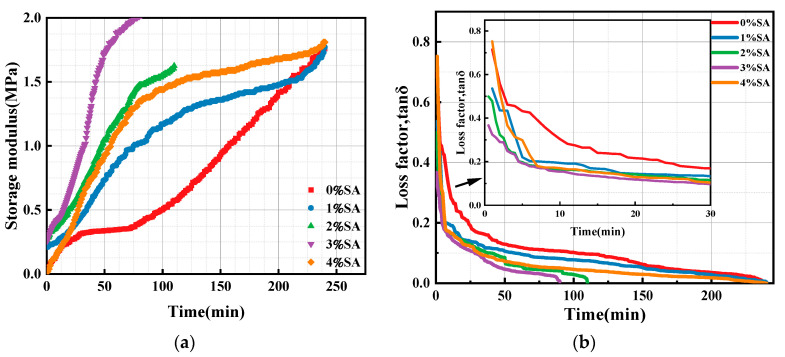
Variation of the storage modulus of SSC with different SA: (**a**) storage modulus; (**b**) loss factor (tan δ).

To explore the relationship between the early structure formation of SSC and time, the storage modulus (G′) of paste was tested, as shown in Figure 9. The G′ reflects the amount of energy stored by the paste when it is deformed under the action of an external force, while the loss factor (tan δ = G″/G′) characterizes the energy dissipation behavior.

As illustrated in Figure 9, the G′ of SSC without SA gradually increased in three stages. In the initial stage, G′ rapidly increased while tan δ declined. During this process, the particles in the paste reached an equilibrium position as a result of the interaction of various forces. Meanwhile, a continuous network of particle interactions began to form, transitioning the paste from a fluid to a solid state [38]. Consequently, the system exhibited a higher G′ and a lower tan δ. In the second stage, both G′ and tan δ exhibited more gradual variation. In the SSC system without SA, both gypsum and Ca(OH)_2_ exhibited low solubility, leading to a reduced alkalinity in the solution. This lower pH significantly decelerated the dissolution rate of GGBFS, enabling the paste to maintain a stable condition for an extended duration. During the third stage, the G′ value progressively increased, whereas the tan δ value continued to decrease. As the particles gradually disintegrated within the paste solution, the ion concentration in the system rose. Meanwhile, the ongoing generation of hydration phases contributed to the solidification of paste, ultimately facilitating its transformation into a more stable and solid structure.

Compared with SSC without SA, the G′ and tan δ of SSC with 1–3% SA changed more rapidly and significantly with increasing SA content [39]. The addition of SA facilitated the dissolution of GGBFS by increasing alkalinity. Furthermore, the amorphous Al(OH)_3_ supplied by SA has the potential to react with Ca^2+^ and SO_4_^2−^ in the solution to form AFt. This reaction facilitates the solution of gypsum and accelerates the formation of hydration products [40]. As SA content increased, G′ continuously increased, tan δ continuously decreased, and the paste cured at a significantly increased rate.

However, the change rates of G′ and tan δ of the SSC with 4% SA did not exhibit a continued increase with higher SA doping. While G′ continued to rise, starting from a lower value, tan δ decreased sharply during the first 10 min and then exhibited a gradual decrease. These findings are in agreement with the yield stress and viscosity results of SSC. Excessive SA can result in diminished rheological characteristics of SSC, which is also damaging to the structural development of SSC. When the dosage of SA reaches a threshold, it causes an increase in the concentration of Al(OH)_4_^−^ present in the solution. Al(OH)_4_^−^ reacted with Ca^2+^ to form a deposit that was captured on the raw material particles, thereby increasing the particle size. In addition, the precipitation process hindered the dissolution of the feedstock, which in turn slowed the curing rate of the paste and resulted in a deceleration in the observed growth in G′.

### 3.2. Compressive Strength

**Figure 10:**
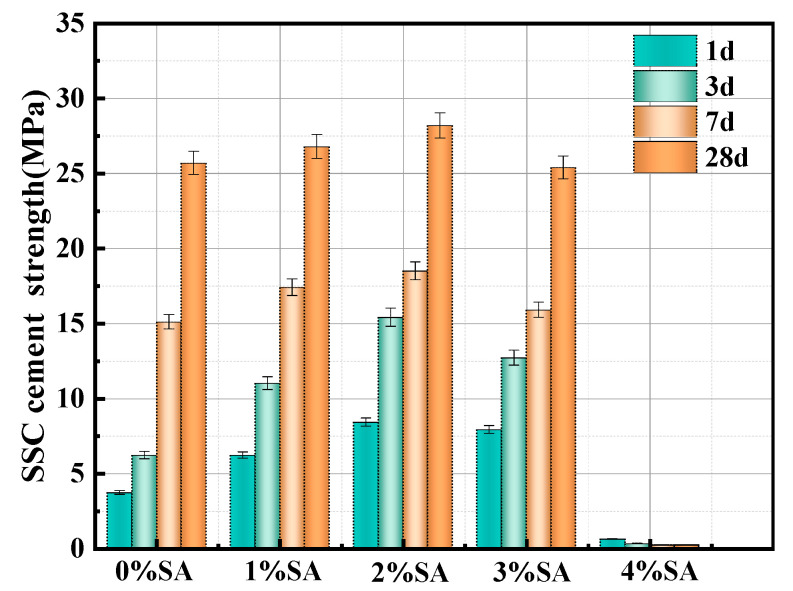
Compressive strength of specimens added SA.

Figure 10 illustrates the influence of different SA dosages on the compressive strength of SSC at 1, 3, 7, and 28 days. This figure clearly shows that an adequate dosage of SA significantly improves the early mechanical performance of SSC. The SSC compressive strengths at 1d, 3d, and 7d were only 3.7, 6.2, and 15.1 MPa, respectively. As the proportion of SA grew, the compressive strength of SSC initially rose and subsequently declined. When the SA content reaches 2%, the 28-day compressive strength of the material attains its maximum level. Compared to SSC without SA, the compressive strength at 1 day, 3 days, and 7 days increased by 127%, 148%, and 22.5%, respectively. However, if the amount of SA exceeds 2%, the compressive strength of the SSC will decrease. Especially when the dosage of SA reaches 4%, SSC is difficult to solidify and almost loses its mechanical properties.

### 3.3. Hydration Heat

**Figure 11:**
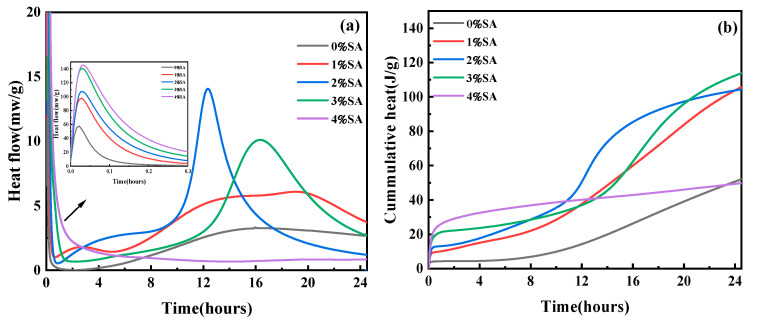
Effect of SA on the hydration heat of SSC: (**a**) hydration heat release curves of the paste; (**b**) cumulative heat release of specimens.

The early performance of SSC is dominated by the hydration reaction. Figure 11a,b show the effects of SA on both the exothermic rate and cumulative exothermic heat of SSC paste.

As illustrated in Figure 11, the incorporation of SA markedly accelerated the exothermic reaction rate and increased the total exothermic heat. Initially, within the first 30 min of the hydration process, an exothermic peak was detected, which can likely be attributed to the heat-releasing dissolution of raw materials like Ca(OH)_2_ [41]. The exothermic peak became more pronounced as the amount of SA increased, suggesting that the hydration reaction was initiated at an early stage. This observation was further corroborated by the increase in early viscosity, as shown in Figure 8.

Approximately 4 h later, the second exothermic peak emerged. This phenomenon is mainly due to the incorporation of SA, leading to an elevated pH level. This, in turn, expedited the dissolution and hydration processes of GGBFS [42]. Among these samples, the SSC containing 2% SA exhibited the highest exothermic rate at approximately 8 h, after which the exothermic rate began to decline around 1 h later. It can be inferred that increasing the amount of SA increases the formation rate of AFt, which is accompanied by a large amount of water consumption, resulting in reduced fluidity in the first hour [43]. In the SSC containing 3% SA, the hydration exothermic rate slowed down, and the heat release curve shifted. However, the cumulative heat release of the SSC with 3% SA was found to exceed that of the other groups. This phenomenon can be explained by the transformation of AFt into AFm, a process that releases extra water and consequently enhances the hydration reaction.

However, the second hydration exothermic peak of SSC with 4% SA was almost absent. This occurrence can be explained by two key reasons. First, the addition of SA provides a significant level of Al(OH)_4_^−^ that reacts with Ca^2+^ and SO_4_^2−^ to form plate-like AFm. Second, the interaction between Al(OH)_4_^−^ and Ca^2+^ promotes the development of hydrated calcium aluminate. This compound forms precipitates on the surface of the raw material, which is a key factor in obstructing the continued hydration process [44]. The coating effect ultimately reduced the exothermic rate during the late hydration phase. Overall, excessive SA impeded the progression of the reaction.

### 3.4. Ion Concentration and pH in Solution

**Figure 12:**
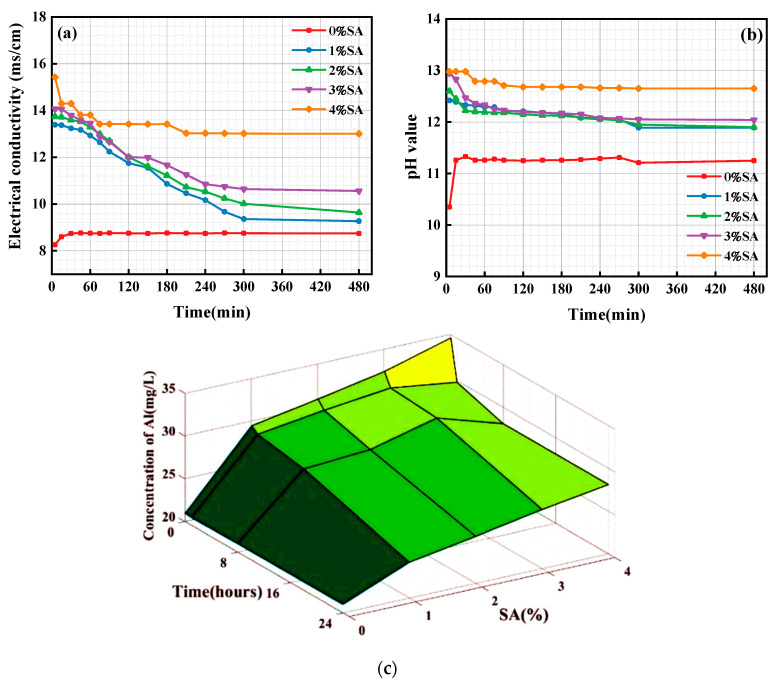
Conductivity, pH value, and Al^3+^ concentration of the pore solution: (**a**) the conductivity of the pore solution; (**b**) the pH value of the pore solution; (**c**) the Al^3+^ concentration in the pore solution.

The development of SSC hydration products is strongly associated with the ionic concentration within the pore solution. These include the generation of the primary early-stage hydration product, AFt, which is influenced by OH^−^ and Al^3+^. Therefore, the conductivity, pH, and Al^3+^ concentration of the pore solution were measured, as illustrated in Figure 12.

As illustrated in Figure 12a, the conductivity of the SSC pore solution without SA initially exhibited a gradual increase and then leveled off around 10 min from the beginning. This indicates that the SSC without SA initiated the dissolution of the raw material to release ions upon contact with water. Once the ion concentration reached a specific threshold, the hydration reaction proceeded continuously, maintaining a state of equilibrium. This is due to the slow dissolution rate of Ca(OH)_2_ due to its limited solubility in the raw material. In an alkaline environment with a pH value of approximately 11, the GGBFS undergoes further dissolution [45]. Gypsum also released Ca^2+^ and SO_4_^2−^, which formed AFt in an alkaline environment. Simultaneously, the dissolution of GGBFS released ions that facilitated the generation of C-(A)-S-H gel, thereby promoting hardening and solidification of SSC [46].

The conductivity of solution in SSC samples containing 1% SA, 2% SA, and 3% SA decreased rapidly in the initial phase before stabilizing. This phenomenon suggested that the incorporation of SA increased the overall ion concentration in the solution. The decrease in solution conductivity indicated that ion consumption during the hydration reaction exceeded the dissolution rate of the raw material, thereby continuously promoting the reaction.

Figure 12b demonstrates the variation in the pH of the SSC solution over time. In the absence of SA, the pH initially increased and then tended to stabilize, as the alkalinity of the solution primarily originates from Ca(OH)_2_. Its dissolution process initially absorbs water and subsequently releases ions, exhibiting characteristics of low solubility and slow dissolution. The pH of the solution was found to be less than that of a saturated solution of Ca(OH)_2_. This may be attributed to the gypsum and GGBFS release of Ca^2+^, thereby inhibiting the dissolution of calcium hydroxide.

However, when SA was added to the SSC system, the pH value initially decreased and then stabilized. Compared with the system without SA, the pH value increased significantly. The pH values of the SSC solutions containing 1% SA, 2% SA, and 3% SA initially decreased slightly and then changed gradually. This is attributed to the fact that SA increased the initial pH value and accelerated the dissolution rate of GGBFS. Subsequently, the amorphous Al(OH)_3_ reacted with dissolved raw materials to form AFt, consuming OH^−^ and thereby promoting the continuous hydration reaction.

The present study aims to further examine the impact of SA on the hydration of SSC, and an Al^3+^ concentration test was conducted. As illustrated in Figure 12c, the Al^3+^ in the SSC without SA primarily originated from the dissolution of GGBFS, and its variation over time was minimal due to the gradual dissolution of GGBFS in the system. In contrast, the Al^3+^ concentration in the SSC solutions containing 1% SA, 2% SA, and 3% SA exhibited a more pronounced initial decrease, followed by a slow decline. This indicates that SA has a substantial influence on the SSC hydration reaction. The increased consumption of Al^3+^ as SA content rises suggests the rapid development of hydration phases. Conversely, in the SSC solution containing 4% SA, the Al^3+^ concentration significantly decreased at the beginning of the reaction but then stabilized at a relatively constant level. The Al^3+^ concentration in the SSC containing 4% SA decreased significantly during the initial phase of the reaction and then remained relatively stable, indicating that SA was involved in the initial hydration reaction, after which the reaction rate slowed down [47]. In summary, the presence of SA in the SSC promotes the early stages of hydration by increasing ion concentration. However, excessive SA (such as in the SSC with 4% SA) may lead to premature stabilization, hindering further hydration.

### 3.5. XRD

**Figure 13:**
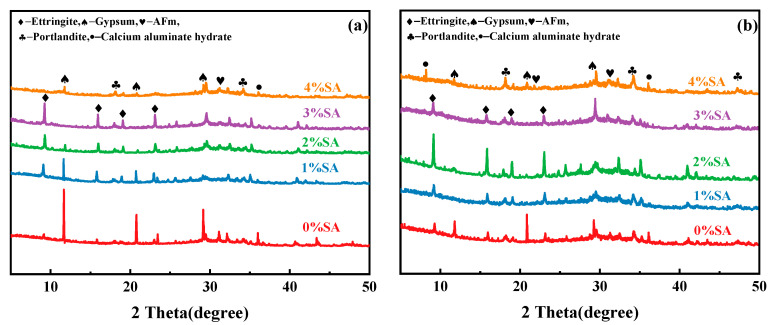
XRD patterns of SSC cement: (**a**) XRD pattern of SSC samples hydrated for 8 h; (**b**) XRD pattern of SSC samples hydrated for 1 day.

The influence of SA on SSC hydration products over time was investigated using XRD. Figure 13 shows the phase compositions of the paste at different SA dosages. Specifically, Figure 13a presents the XRD pattern of SSC hydration after 8 h, while Figure 13b shows the XRD pattern after 1 day.

After hydrating the specimens for 8 h, diffraction peaks indicative of AFt were detected in the SSC samples both in the absence of SA and in the presence of 1%, 2%, and 3% SA (as illustrated in Figure 13a). The XRD patterns of the SSC samples without SA and those containing 1% SA and 2% SA exhibited diffraction peaks for gypsum, while no gypsum diffraction peaks were detected in the SSC sample with 3% SA. This observation indicates that the 3% SA sample reacts more rapidly in the system, leading to greater consumption of gypsum.

Moreover, as the concentration of SA increased, the intensity of AFt peaks also rose. This suggests a positive correlation between SA content and AFt formation. This phenomenon may be attributed to SA releases of Al(OH)_3_ and OH^−^ into the solution (the formation process is shown in Equation (4)). This process accelerated the dissolution of raw materials and facilitated AFt generation in the system. However, in the SSC with 4% SA, although gypsum and Ca(OH)_2_ phases were present, no AFt generation was observed. This phenomenon may be attributed to the products generated from excess aluminum salts that coat the raw materials and prevent the hydration reaction process (the reaction is shown in Equation (8)) [48].

NaAlO_2_ + H_2_O ⇄ Al(OH)_3_ + NaOH (4)

3SO_4_^2−^ + 2Al(OH)_3_ + 6Ca^2+^ + 6OH^−^ + 26H_2_O → 3AFt (5)

AFt + 4Al(OH)_4_^−^ + 6Ca^2+^ + 8OH^−^ → 3AFm + 8H_2_O (6)

Ca(OH)_2_ + Al(OH)_3_ + CaSO_4_ → AFm (7)

4Ca(OH)_2_(aq) + 2Al(OH)_4_^−^ (aq) → Ca_4_Al_2_(OH)_14_(s) + 2OH^−^(aq) (8)

When the hydration duration was 1 day (Figure 13b), the gypsum diffraction peaks of the SSC samples without SA and with 1% SA decreased compared to those at the 8 h hydration period, while the AFt peak intensity increased. This shows that the SSC system was undergoing a continuous hydration process, leading to increased AFt formation. In contrast, the SSC with 4% SA showed no new hydration products as the hydration time increased, with only minor changes in the gypsum and Ca(OH)_2_ diffraction peaks. The SSC with 3% SA showed a significant decrease in AFt peak intensity compared to the 8 h group, suggesting that the excess aluminate may have transformed AFt into AFm after its formation, as illustrated in Equations (5)–(7) [49,50]. Furthermore, the premature generation of AFt results in an accelerated consumption of water molecules, accelerating hardening and impeding subsequent hydration of the specimens. Figure 13b also shows that the gypsum diffraction peaks of the SSC with 1% SA and 2% SA significantly decreased. Conversely, the AFt diffraction peak intensity for the SSC with 2% SA was higher than that of the SSC with 1% SA and 3% SA. This observation suggests that continuous AFt formation is more favorable when the SA content is 2%.

The analysis indicates that the incorporation of SA into SSC promotes the early generation of hydration phases such as AFt in the system. However, it is crucial to keep the SA dosage within an optimal range to ensure effective hydration.

### 3.6. TG-DTG

**Figure 14:**
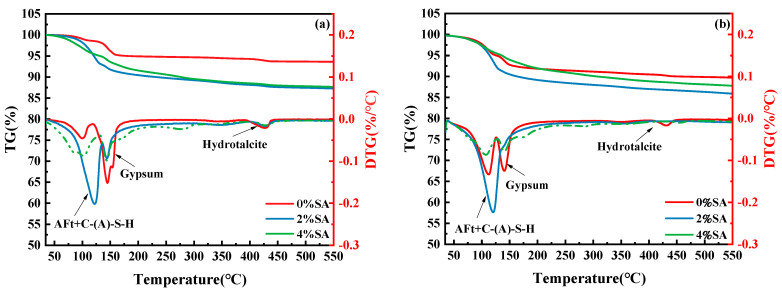
TG-DTG curves of hydrated paste for 8 h and 1 day: (**a**) thermogravimetric curves of samples hydrated for 8 h; (**b**) thermogravimetric curves of samples hydrated for 1 day.

TG analysis was performed on SSC samples with 0%, 2%, and 4% SA at both 8 h (Figure 14a) and 1 day (Figure 14b) to analyze the impact of SA on the hydration products in SSC. As illustrated in Figure 14, the TG curve of SSC displays two prominent weight loss peaks. The initial peak observed between 50 °C and 140 °C is attributed to the removal of bound water from AFt and C-(A)-S-H gel [51]. The second peak can be attributed to the dehydration of gypsum, which occurs with a temperature range of 110 °C to 150 °C [52].

As illustrated in Figure 14a, compared with the SSC without SA, the 2% SA accelerated AFt formation in the 8 h sample, resulting in a significant increase in gypsum consumption. Meanwhile, the weight loss peaks associated with AFt and C-A-S-H gel increased. However, when the content of SA reached 4%, the weight loss peaks of hydration products like AFt decreased. The peak of the AFm phase appeared at 180–200 °C, and the peak of the Al(OH)_3_ dewatering appeared at 225–300 °C, indicating that excessive SA promoted the generation of AFm. Furthermore, the weight loss peaks of Ca(OH)_2_ exhibited only slight changes within the temperature interval from 375 to 440 °C. This variation indicated that the calcium consumed for the early generation of hydration phases might have been sourced from more soluble raw materials [53].

As illustrated in Figure 14b, the weight loss peaks for AFt and C-(A)-S-H gel in SSC with 2% SA were notably higher after 1 day than those in SSC without SA, whereas the weight loss peak for gypsum showed a decrease. This indicates that SA accelerates the formation of AFt in SSC. In SSC with 4% SA, the peaks for C-(A)-S-H gel and the Al(OH)_3_ phase continuously decreased, while the AFm weight loss peak continuously increased, indicating that excess aluminum salts promote the formation of AFm. Additionally, incorporating SA into the system enhances the consumption of Ca(OH)_2_ in the initial hydration phase [54]. There was still gypsum remaining after 1d In SSC with 4% SA, which corresponded to XRD analysis results; 4% SA produces AFm, which is wrapped in GGBFS and gypsum surface, hindering hydration reaction and causing the strength development to be affected. Therefore, it can be inferred that excess SA not only inhibits the hydration of SSC but also promotes the formation of AFm.

### 3.7. SEM Analysis

Samples of 0% SA, 2% SA, and 4% SA were analyzed by SEM after 1 day of hydration to investigate the impact of SA on the distribution and generation of hydration phases in the SSC system. The lamellar structure of the 4% SA sample was analyzed using EDS points.

**Figure 15:**
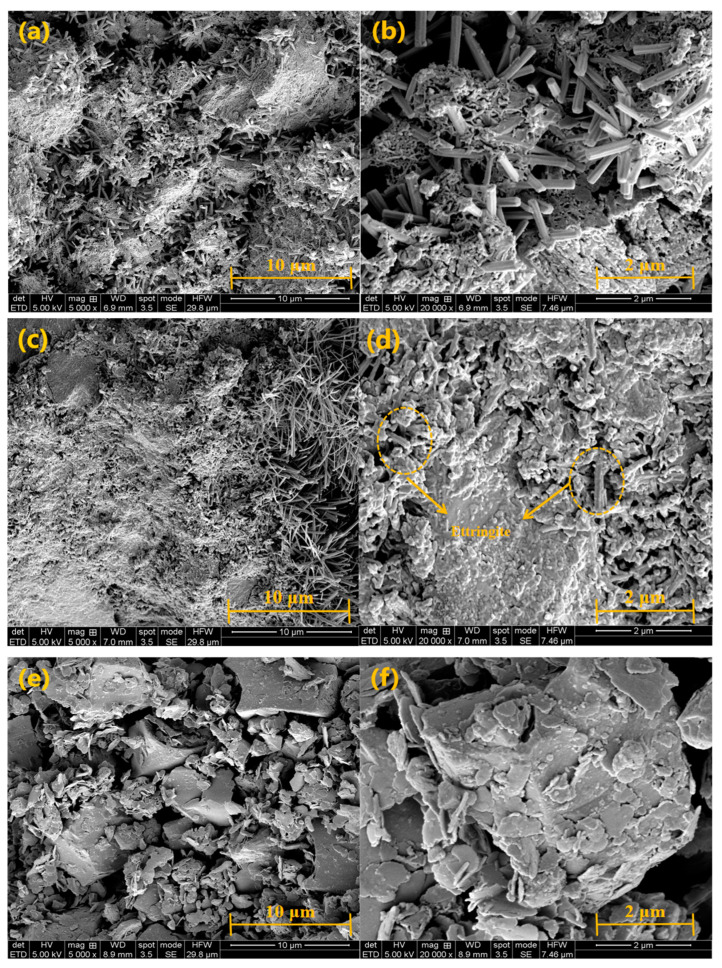
SEM images of samples 0% SA (**a**,**b**), 2% SA (**c**,**d**), and 4% SA (**e**,**f**) at 1 day.

As shown in Figure 15, the microstructure of samples from the SSC without SA after 1 day of hydration exhibits a combination of needle-like and gel-like hydration products. The SSC without SA showed pronounced pores and a relatively loose structure, with AFt products between the gels mainly showing a short columnar morphology. In contrast, the SSC with 2% SA displayed a denser structure, with AFt mostly exhibiting a needle-like morphology and C-(A)-S-H gel, characterized by overlapping growth patterns. Additionally, the needle-like hydration products displayed uniform orientation and regular growth [55], suggesting a more organized and structured development of hydration phases. However, in the SSC with 4% SA, numerous undissolved GGBFS particles were observed, with plate-like products stacked on top of the particles. Notably, no needle-like hydration phases were present. This is consistent with the results shown in Figure 13, where no AFt products were detected. The flake-like structure is more likely to correspond to AFm or hydrated calcium aluminate.

**Figure 16:**
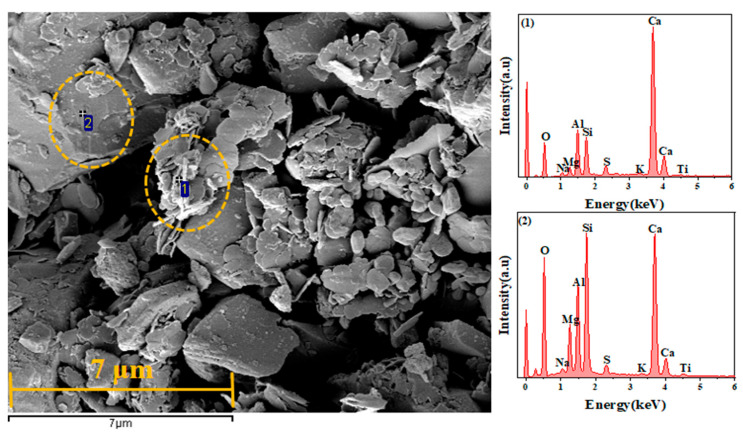
SEM-EDS pictures of SSC with 4% SA at 1 day.

As illustrated in Figure 16, the EDS point analysis was conducted on the SSC with 4% SA. The Region 1 analysis revealed that the region mainly comprised lamellar hydration products, with higher concentrations of calcium and aluminum and relatively lower concentrations of silicon. This composition indicated that this region was the site where AFm and hydrated calcium aluminate were formed, both of which exhibit a lamellar structure. These hydration products encapsulated the raw material particles, which effectively prevented further hydration of GGBFS [56]. This observation is consistent with previous experimental findings. Additionally, the combination of SEM morphology and element distribution in Region 2 further supports the presence of unhydrated GGBFS particles in this area. In summary, moderate SA incorporation promotes the SSC hydration reaction, while excessive SA inhibits the hydration reaction and promotes the formation of AFm.

### 3.8. Degrees of GGBFS Hydration

**Figure 17:**
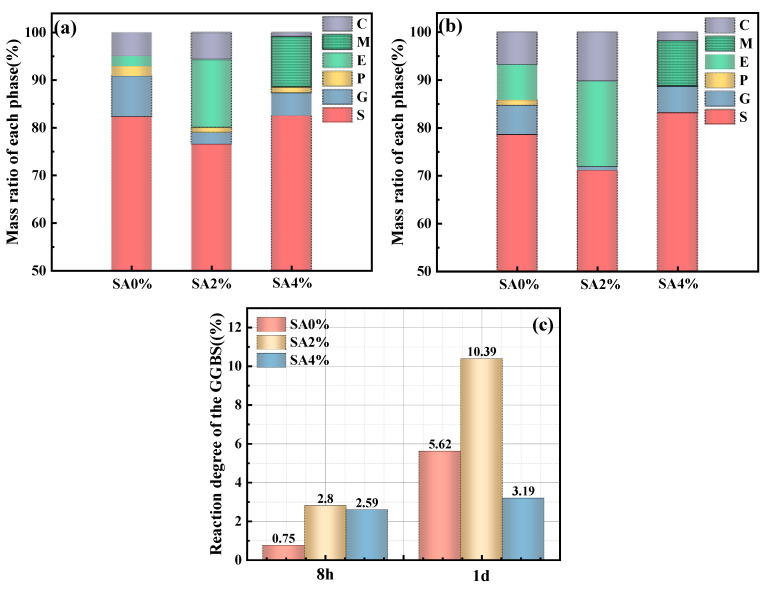
Phase assemblages and GGBFS reaction degree (S: GGBFS; G: gypsum; P: Portlandite; E: ettringite; M: AFm; C: C-(A)-S-H): (**a**) the phase assemblages of samples at 8 h; (**b**) the phase assemblages of samples at 1 day; (**c**) the GGBFS hydration degree.

The hydration degree of GGBFS at 8 h and 1 day was calculated, and the SSC powdered samples were analyzed using EDTA dissolution. Figure 17a,b illustrate the 8 h and 1 day changes in the phase components. Figure 17c depicts the GGBFS hydration degree over time.

As shown in Figure 17a,b, gypsum in the SSC with 2% SA was almost completely consumed within 1 day. In contrast, the consumption of gypsum in the SSC with 4% SA was significantly slower than in the without SA, indicating that an appropriate amount of SA facilitated the consumption of gypsum. The changes in C-(A)-S-H in relation to the amount and duration of SA in that system further highlight the effect of SA on hydration product formation. Figure 17c shows that the GGBFS hydration level in the SSC with 2% SA was much higher than in the SSC without SA after 1 day. However, the GGBFS hydration process in the SSC with 4% SA was delayed, emphasizing the crucial role of the optimal amount of SA in facilitating the hydration of GGBFS.

**Figure 18:**
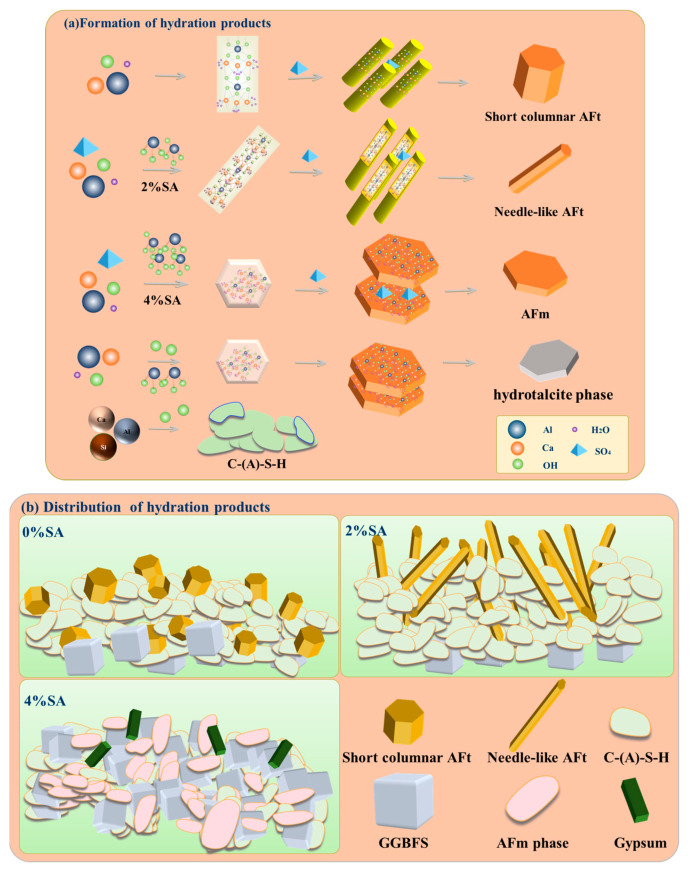
Formation and distribution of SSC hydration products: (**a**) formation of hydration products; (**b**) distribution of hydration products.

As shown in Figure 18a,b, raw material showed a slow dissolution rate in the SSC system without SA, leading to low Al concentration in the pore solution. Resulting in the formation of AFt was prevented, which predominantly developed into short columnar structures. The limited amount of short columnar AFt is not conducive to effective bonding with C-(A)-S-H, ultimately resulting in slower development of macroscopic strength. SA releases OH^−^ and Al(OH)_3_ [57]. OH^−^ improves the SSC system’s alkalinity and accelerates the GGBFS dissolution. The amorphous aluminum phase reacts with SO_4_^2−^, Ca, and Si to form AFt and C-(A)-S-H gel. Because of the high Al concentration in the system, the main morphology of AFt is needle-like. The early property of the SSC is increased because the acicular AFt can better bind to the C-(A)-S-H gel. However, the SSC with 4% SA did not exhibit an AFt phase. Instead, the resulting product was primarily the AFm phase. The AFm phase is mainly Ca_2_Al(OH)_6_·X·xH_2_O, where a Ca_2_Al(OH)_6_ is a cationic layer, and X represents an interlayer anion, which is replaced by an anion radical, such as OH^−^, SO_4_^2−^, and CO_3_^2−^. The structure of this hydration product is similar to that of hydrated calcium aluminate, where the interlayer anion is typically OH^−^, and both share a hexagonal plate-like structure [58]. The residual gypsum in the SSC with 4% SA indicates that the formed product coats the interface of the raw material, adsorbing anions and thus preventing raw material dissolution and subsequent hydration action. Above all, a moderate addition amount of SA enhances the extent of GGBFS hydration, while an excessive amount of SA prevents the hydration reaction of GGBFS. The optimal dosage of SA is 2%.

## 4. Conclusions

In this study, the effects of SA on the setting time, rheological performance, and hardening behavior of the SSC system were investigated, and the hydration mechanism was explored. The conclusions are drawn as follows:

(1) SA increased the viscosity of SSC and promoted early-stage structural development, stabilized and solidified the paste, and shortened the setting time, as determined by rheological properties and setting time tests. However, excessive SA retarded the hardening process and prolonged the setting time;
(2) The early compressive strength of SSC is significantly enhanced when an appropriate amount of SA. Specifically, when the SA content is 2%, the 1-day compressive strength increases by 127%, reaching up to 8.7 MPa. However, the compressive strength of SSC decreased when the SA content reached 4%;
(3) SA not only raised the pH value of the SSC system but also functioned as a source of aluminum. This dual role enhanced the dissolution of GGBFS and facilitated the formation of AFt and C-(A)-S-H gel. An appropriate amount (2%) of SA transformed AFt from a short columnar to a needle-like morphology. This transformation enhanced the compatibility and microstructure of the crystal-gel network, ultimately leading to improved mechanical properties;
(4) Excessive SA led to the generation of an AFm phase that coats the surface of the raw materials, consequently hindering the hydration process and playing an adverse role in the mechanical properties of SSC.

This study focused on the early-stage performance of SSC with SA, the long-term strength development, and durabilities, such as carbonation resistance and freeze–thaw resistance, which remain to be investigated. Long-term exposure tests and monitoring of SSC specimens with different SA dosages could provide valuable insights into the long-term durability and reliability of SSC in practical engineering applications.

## Figures and Tables

**Table 1:** Chemical composition of materials (wt%).

	CaO	SiO_2_	Al_2_O_3_	Fe_2_O_3_	MgO	K_2_O	SO_3_	TiO_2_	Others
GGBFS	34.46	32.63	16.90	0.26	10.56	0.35	2.37	0.69	1.78
Gypsum	37.18	2.10	0.74	0.40	1.13	0.14	44.79	0.03	13.49

## Data Availability

The original contributions presented in this study are included in the article, and further inquiries can be directed to the corresponding authors.

## References

[1] Cabrera-Luna K., Maldonado-Bandala E.E., Nieves-Mendoza D., Escalante García J.I. (2018). Supersulfated binders based on volcanic raw material: Optimization, microstructure and reaction products. Constr. Build. Mater..

[2] Erdem E., Ölmez H. (1993). The mechanical properties of supersulphated cement containing phosphogypsum. Cem. Concr. Res..

[3] Yu B., Fang Z., Gao Y., Yang W., Wang C., Zhou S. (2023). Carbonation of supersulfated cement concrete after 8 years of natural exposure. Cem. Concr. Compos..

[4] Wu Q., Xue Q., Yu Z. (2021). Research status of super sulfate cement. J. Clean. Prod..

[5] Cabrera-Luna K., Maldonado-Bandala E.E., Nieves-Mendoza D., Castro-Borges P., García J.I.E. (2020). Novel low emissions supersulfated cements of pumice in concrete; mechanical and electrochemical characterization. J. Clean. Prod..

[6] Pinto S.R., Angulski da Luz C., Munhoz G.S., Medeiros-Junior R.A. (2020). Resistance of phosphogypsum-based supersulfated cement to carbonation and chloride ingress. Constr. Build. Mater..

[7] Chen H., Song Z., Liu B., Sun G., Hou P., Li Q., Wang Y., Zhang P., Cheng X. (2025). Improving the carbonation resistance of supersulfated cement by nano SiO2 and silica fume. Cem. Concr. Compos..

[8] Xi X., Zheng Y., Zhuo J., Zhang P., Golewski G.L., Du C. (2024). Mechanical properties and hydration mechanism of nano-silica modified alkali-activated thermally activated recycled cement. J. Build. Eng..

[9] Golewski G.L. (2024). Determination of Fracture Mechanic Parameters of Concretes Based on Cement Matrix Enhanced by Fly Ash and Nano-Silica. Materials.

[10] Deng X.F., Li M.g., Wang Y.F., Wang J.T., Zhang J.J., Yang Z.W., He X.Y., Yang J., Tan H.B. (2024). Impact of ettringite seeding on hydration, strength and shrinkage of Na_2_SO_4_ activated slag. Compos. Part B.

[11] Tambara Júnior L.U.D., de Matos P.R., Lima G.S., Silvestro L., Rocha J.C., Campos C.E.M., Gleize P.J.P. (2022). Effect of the nanosilica source on the rheology and early-age hydration of calcium sulfoaluminate cement pastes. Constr. Build. Mater..

[12] Rubert S., Angulski da Luz C., Varela M.V.F., Pereira Filho J.I., Hooton R.D. (2018). Hydration mechanisms of supersulfated cement. J. Therm. Anal. Calorim..

[13] Xing J.R., Zhou Y., Peng Z.C., Wang J.W., Jin Y.J., Jin M. (2023). The influence of different kinds of weak acid salts on the macro-performance, micro-structure, and hydration mechanism of the supersulfated cement. J. Build. Eng..

[14] Huang J.Y., Huang J., Min J.j., Lv R.Y., Kuang H., Hu H.L., Yang R., Tang P., Zhao Q.L., Jian S.W. (2024). Hydration mechanism of a sodium-doped phosphogypsum-based hemihydrate whisker (omongwaite) in supersulfated cement system. Constr. Build. Mater..

[15] De Belie N., Grosse C.U., Kurz J., Reinhardt H.W. (2005). Ultrasound monitoring of the influence of different accelerating admixtures and cement types for shotcrete on setting and hardening behaviour. Cem. Concr. Res..

[16] Wang H.C., Feng P., Liu X., Shi J.S., Wang C., Wang W., Li H., Hong J.X. (2024). The role of ettringite seeds in enhancing the ultra-early age strength of Portland cement containing aluminum sulfate accelerator. Compos. Part B.

[17] Zhang G.T., Li M.Q., Zhu Z.Y. (2023). Effect of Aluminium Substitution on Physical Adsorption of Chloride and Sulphate Ions in Cement-Based Materials. Materials.

[18] Chang N., Li H., Liu W.H., Zheng W.K., Zhu H.M., Wan Z.M., Wu X.Z., Jiang H.J., Zhang L. (2024). Improved macro-microscopic characteristic of gypsum-slag based cementitious materials by incorporating red mud/carbide slag binary alkaline waste-derived activator. Constr. Build. Mater..

[19] Li H., Xu F., Li B., Sun T., Huang X.M., Zhu J., Peng C., Lin J.t., Chen Z.W. (2022). Investigation on mechanical properties of excess-sulfate phosphogypsum slag cement: From experiments to molecular dynamics simulation. Constr. Build. Mater..

[20] Gracioli B., Angulski da Luz C., Beutler C.S., Pereira Filho J.I., Frare A., Rocha J.C., Cheriaf M., Hooton R.D. (2020). Influence of the calcination temperature of phosphogypsum on the performance of supersulfated cements. Constr. Build. Mater..

[21] Kang C., Kim T. (2022). Effect of reverse-osmosis brine and sodium aluminate on the hydration properties and strength of alkali-activated slag cement. Case Stud. Constr. Mater..

[22] (2011). Test Methods for Water Requirement of Normal Consistency, Setting Time and Soundness of the Portland cement.

[23] (2021). Test method of cement mortar strength.

[24] Liu H., Jing W., Qin L., Duan P., Zhang Z., Guo R., Li W. (2022). Thermal stability of geopolymer modified by different silicon source materials prepared from solid wastes. Constr. Build. Mater..

[25] Luan C., Yang Q., Lin X., Gao X., Cheng H., Huang Y., Du P., Zhou Z., Wang J. (2023). The synergistic effects of ultrafine slag powder and limestone on the rheology behavior, microstructure, and fractal features of ultra-high performance concrete (UHPC). Matersial.

[26] Mukherjee S., Kumar R., Behera M., Goyal A., Rahman M.R.J.D.i.t.B.E. (2025). Rheology, mechanical properties and microstructure characterization of limestone calcined clay cement (LC3) incorporated sustainable lightweight self-compacting concrete. Dev. Built Environ..

[27] Zhao D., Williams J.M., Park A.-H.A., Kawashima S.J.C., Research C. (2023). Rheology of cement pastes with calcium carbonate polymorphs. Cem. Concr. Res..

[28] Zhang C., Wang J.W., Zhang X.Z., Hou J., Huang J.L., Feng S.X., Wang J.B., Duan G.B. (2024). Influence of limestone powder on water film thickness and plastic viscosity of UHPC. Case Stud. Constr. Mater..

[29] (2023). Standard Examination Methods for Drinking Water—Part 6: Metal and Metalloid Indices.

[30] van Aardt J.H.P., Visser S. (1975). Thaumasite formation: A cause of deterioration of portland cement and related substances in the presence of sulphates. Cem. Concr. Res..

[31] (2019). Quantitative Determination of Constituents of Cement.

[32] Qi G.Z., Zhang Q., Sun Z.N. (2024). Mechanical properties and hydration mechanism of super-sulfated cement prepared with ordinary Portland cement, carbide slag, and sodium silicate. Front. Mater..

[33] Li B.B., Hou P.K., Chen H., Zhao P.Q., Du P., Wang S.D., Cheng X. (2022). GGBS hydration acceleration evidence in supersulfated cement by nanoSiO_2_. Cem. Concr. Compos..

[34] Way S.J., Shayan A. (1989). Early hydration of a portland cement in water and sodium hydroxide solutions: Composition of solutions and nature of solid phases. Cem. Concr. Res..

[35] Zarzuela R., Luna M., Carrascosa L.M., Yeste M.P., Garcia-Lodeiro I., Blanco-Varela M.T., Cauqui M.A., Rodríguez-Izquierdo J.M., Mosquera M.J. (2020). Producing C-S-H gel by reaction between silica oligomers and portlandite: A promising approach to repair cementitious materials. Cem. Concr. Res..

[36] Zuo Y.B., Ye G. (2020). Preliminary Interpretation of the Induction Period in Hydration of Sodium Hydroxide/Silicate Activated Slag. Materials.

[37] Gou M.F., Hou W.L., Zhou L.F., Zhao J.H., Zhao M.K. (2023). Preparation and properties of calcium aluminate cement with Bayer red mud. Constr. Build. Mater..

[38] Mostafa A.M., Yahia A. (2016). New approach to assess build-up of cement-based suspensions. Cem. Concr. Res..

[39] Zhang C., Zhang X.Z., Hou J., Wang J.W., Duan G.B. (2022). Rheology and early microstructure evolution of fresh ultra-high performance concrete with polycarboxylate superplasticizer. Case Stud. Constr. Mater..

[40] Maach N., Georgin J.F., Berger S., Pommay J. (2021). Chemical mechanisms and kinetic modeling of calcium aluminate cements hydration in diluted systems: Role of aluminium hydroxide formation. Cem. Concr. Res..

[41] Wang L., Gao Z.Y., Gao F.H., Li X.Y., Chang S., Liu S.H. (2024). Comparing study on the evolution characteristics of performance and microstructure between Portland slag cement and supersulfated cement under chemical attacks. Constr. Build. Mater..

[42] Blotevogel S., Doussang L., Poirier M., André L., Canizarès A., Simon P., Montouillout V., Kaknic J., Patapy C., Martin C. (2024). The influence of Al_2_O_3_, CaO, MgO and TiO_2_ content on the early-age reactivity of GGBS in blended cements, alkali-activated materials and supersulfated cements. Cem. Concr. Res..

[43] Herrera-Mesen C., Salvador R.P., Cavalaro S.H.P., Aguado A. (2019). Effect of gypsum content in sprayed cementitious matrices: Early age hydration and mechanical properties. Cem. Concr. Compos..

[44] Paglia C., Wombacher F., Böhni H. (2001). The influence of alkali-free and alkaline shotcrete accelerators within cement systems: I. Characterization of the setting behavior. Cem. Concr. Res..

[45] Garcia-Lodeiro I., Palomo A., Fernández-Jiménez A., Macphee D.E. (2011). Compatibility studies between N-A-S-H and C-A-S-H gels. Study in the ternary diagram Na_2_O–CaO–Al_2_O_3_–SiO_2_–H_2_O. Cem. Concr. Res..

[46] Ouyang G.S., Li Z.W., Sun T., Ye Z.Y., Deng Y.Y., Li W.T. (2024). Greener phosphogypsum-based all-solid-waste cementitious binder with steel slag activation: Hydration, mechanical properties and durability. J. Clean. Prod..

[47] Burris L.E., Kurtis K.E. (2018). Influence of set retarding admixtures on calcium sulfoaluminate cement hydration and property development. Cem. Concr. Res..

[48] Ye X.F., Zhao X.D., Ming Q., Zhu J., Guo J.M., Sun D.Q., Zhang S., Xu J., Zhou Z. (2021). Process optimization to enhance utilization efficiency of precipitants for chloride removal from flue gas desulfurization wastewater via Friedel’s salt precipitation. J. Environ. Manag..

[49] Han J.G., Wang K.J., Shi J.Y., Wang Y. (2014). Influence of sodium aluminate on cement hydration and concrete properties. Constr. Build. Mater..

[50] Christensen A.N., Jensen T.R., Hanson J.C. (2004). Formation of ettringite, Ca_6_Al_2_(SO_4_)_3_(OH)_12_·26H_2_O, AFt, and monosulfate, Ca_4_Al_2_O_6_(SO_4_)·14H_2_O, AFm-14, in hydrothermal hydration of Portland cement and of calcium aluminum oxide—Calcium sulfate dihydrate mixtures studied by in situ synchrotron X-ray powder diffraction. J. Solid State Chem..

[51] Bian Z.W., Jin G.W., Ji T. (2021). Effect of combined activator of Ca(OH)_2_ and Na_2_CO_3_ on workability and compressive strength of alkali-activated ferronickel slag system. Cem. Concr. Compos..

[52] Gijbels K., Pontikes Y., Samyn P., Schreurs S., Schroeyers W. (2020). Effect of NaOH content on hydration, mineralogy, porosity and strength in alkali/sulfate-activated binders from ground granulated blast furnace slag and phosphogypsum. Cem. Concr. Res..

[53] Zhang X.W., Lu C.X., Shen J.Y. (2016). Influence of tartaric acid on early hydration and mortar performance of Portland cement-calcium aluminate cement-anhydrite binder. Constr. Build. Mater..

[54] Li Y., Qiao C.Y., Ni W. (2020). Green concrete with ground granulated blast-furnace slag activated by desulfurization gypsum and electric arc furnace reducing slag. J. Clean. Prod..

[55] Salvador R.P., Cavalaro S.H.P., Segura I., Figueiredo A.D., Pérez J. (2016). Early age hydration of cement pastes with alkaline and alkali-free accelerators for sprayed concrete. Constr. Build. Mater..

[56] Gevers B.R., Labuschagné F.J.W.J. (2020). Green Synthesis of Hydrocalumite (CaAl-OH-LDH) from Ca(OH)_2_ and Al(OH)_3_ and the Parameters That Influence Its Formation and Speciation. Crystals.

[57] Wang L., He Z., Cai X.H. (2011). Characterization of pozzolanic reaction and its effect on the C-S-H Gel in fly Ash-cement paste. J. Wuhan Univ. Technol..

[58] Wang X.G., Zhu J.L., Lei Y.X., Lei W.Y. (2022). Synthesis and characterization of layered double hydroxides hybrid microcapsules for anticorrosion via self-healing and chloride ion adsorption. Appl. Clay Sci..

